# The Challenge of Differentiating Mixed Histiocytosis: A Case Report

**DOI:** 10.1155/crh/7099157

**Published:** 2026-06-18

**Authors:** Lydia Harris, Rosalia Arnolda, David Ogburn, Padmaja Mallidi

**Affiliations:** ^1^ Department of Internal Medicine, Carilion Clinic, Roanoke, Virginia, USA, carilionclinic.org; ^2^ School of Medicine, Virginia Tech, Blacksburg, Virginia, USA, vt.edu; ^3^ Department of Oncology, Carilion Clinic, Roanoke, Virginia, USA, carilionclinic.org

**Keywords:** Erdheim–Chester disease, Langerhans cell histiocytosis, pericardial effusion

## Abstract

Langerhans cell histiocytosis (LCH) and Erdheim–Chester Disease (ECD) are two forms of histiocytic disease with some overlapping features. They can have multisystem involvement, which makes it a diagnostic challenge. In this report, we present a patient who was diagnosed with both LCH and ECD over the span of 7 years. A 69‐year‐old female with a past medical history of hypertension, hyperlipidemia, and diabetes mellitus presented with right ear pressure and was subsequently diagnosed with LCH after a mastoid bone biopsy in 2017. She then underwent mass resection. Two years later, during an abdominal MRI for evaluation of cholestatic disease, a large pericardial effusion with pericardial thinning was discovered. Cardiac MRI was concerning for a soft tissue mass anterior and superior to the right atrium, suggestive of infiltrative processes such as histiocytosis. Seven years after the initial presentation, she underwent a pericardial biopsy that diagnosed ECD. It is important to recognize that even though mixed disease is rare, clinical suspicion needs to be high. This is particularly true for patients with cardiac features that would be atypical in LCH to ensure prompt treatment.

## 1. Introduction

Langerhans cell histiocytosis (LCH) and Erdheim–Chester disease (ECD) are the two most common histiocytic diseases [1]. Histiocytes are phagocytic cells that are localized to specific tissues and act as part of the innate immune system [2]. Histiocytic disease occurs when granulomatous lesions arise from the accumulation of these cells and the corresponding inflammatory infiltrate [1]. These lesions can arise in many of the body’s organ systems, predominantly seen in the skin, bones, and lungs [1]. As these lesions can occur in any part of the body and do not arise with a particular pattern, histiocytic disease is frequently a challenge to diagnose clinically.

LCH is the most common form of histiocytic disease and spans all age ranges from within the first year of life to the elderly [1]. Prevalence has been reported as being as high as 9 per 1 million people aged < 1, as high as 4.46 per 1 million aged < 14, and as high as 1.06 per 1 million aged > 15 [3, 4]. LCH is classified by the neoplastic proliferation of myeloid precursor cell differentiation into CD1a+/CD207+/S100 cells [5]. These cells lead to the activation of the immune system in the tissue where this proliferation occurs, leading to the recruitment of eosinophils, neutrophils, lymphocytes, and macrophages. Ultimately, this immune activation consequently results in the formation of eosinophilic granulomas [5]. Like other histiocytic diseases, this pathological process can occur anywhere in the body, with most severe disease being classified as those with lesions in the liver, spleen, or hematopoietic system. LCH is multisystem at the time of diagnosis in two‐thirds of adult patients, further complicating the diagnosis and treatment of the disease [5].

ECD is a much rarer histiocytic disease, with under 1000 cases in the literature [6]. ECD tends to predominantly be observed in patients 55–60, with the disease more commonly observed in male patients (3:1) [2]. Rare cases have also been observed in children [2]. Skeletal involvement is a defining feature of ECD, occurring in almost all patients, with the predominant finding being bilateral symmetric cortical osteosclerosis of the metaphyseal and diaphyseal regions [2]. Of note, cardiovascular involvement is observed in > 50% of patients, and one‐third of patients have retroperitoneal fibrosis involving the kidneys and ureters, CNS involvement, and periorbital xanthelasma [2]. Diagnosis of ECD is commonly made through PET‐CT observation of the aforementioned skeletal findings. For diagnosis, biopsy and histology is required, despite the “foamy mononucleated histiocytes with small nuclei” being a nonspecific finding.

It has been reported that 10%–15% of adults with ECD also have a LCH diagnosis [7]. This rate of combined ECD‐LCH was higher in patients with > 4 sites of active disease and in females [7]. This mixed disease was found to commonly be observed in the long bones, CNS, facial orbits, lungs, hypothalamic‐pituitary axis, and skin [7]. Studies suggest that up to 48% with mixed ECD‐LCH of patients have simultaneous diagnosis, often with histopathological findings of both diseases present in a single biopsy [8]. In patients that were not diagnosed simultaneously, LCH was always found to be diagnosed first, with a median of 13.5 years between diagnoses [8]. Mixed disease has been related to BRAF V600E mutations, leading to the observation of improved clinical outcomes observed in patients treated with BRAF targeted therapies [7]. Thus, it is essential for clinicians to remain cognizant of the relationship between these two histiocytic diseases and to remember that there might be years between diagnosis of LCH and ECD. We present a case of mixed ECD‐LCH that was diagnosed 3 years apart.

## 2. Case

A 69‐year‐old female with a past medical history of hypertension, hyperlipidemia, Type II diabetes mellitus, initially presented in 2017 with right ear pressure that was not relieved after the placement of tympanostomy tubes. Further workup via CT of the orbit and sella showed evidence of pervasive and destructive right‐sided otomastoiditis (Figure 1a), and MRI showed large homogeneously enhancing right temporal bone (Figure 1b). Biopsy of the right mastoid bone initially showed osteomyelitis, and she was treated with a course of antibiotics. Repeat biopsy showed eosinophilic granuloma and unifocal LCH, staining positive for S100 and CD1a, and she underwent resection.

**FIGURE 1:**
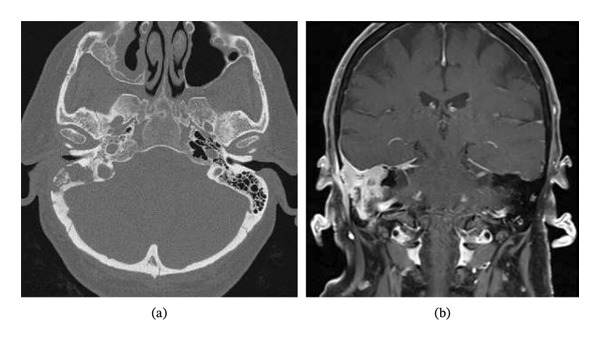
(a) LCH of mastoid bone lesion seen on CT in 2017. Noted to have right‐sided mastoid destruction with multifocal destruction along the posterior aspect of the osseous portion of the external auditory canal and with multifocal tegmen destruction extending into the lower posterior margin of the vestibular aqueduct. Noted to have extensive dehiscence along the posterolateral margin of the mastoid segment of Cranial Nerve VII with deossification inferolateral to the tympanic segment of Cranial Nerves VII. (b) LCH of mastoid bone lesion on MRI. Obtained in 2017. MRI with more detailed characterization of homogeneously enhancing right temporal bone mass which destroys multiple portions of the posterior wall of the osseous portion of the right external auditory canal, right sigmoid plate, and extensive portions of the right tegmen tympani. This process pervades the right aditus ad antrum and right middle tympanic cavity. Destruction also presents along the lateral aspect of the mastoid process with tissue extending laterally through that vicinity. There is also destruction about the mastoid segment of Cranial Nerve VII. Based on MRI, the findings are more concerning for tumor rather than postobstructive fluid or osteomyelitis.

In 2019, she had symptoms concerning for carotid stenosis and was hospitalized. A CT of the head and neck revealed 80% focal stenosis of the left carotid artery and moderate stenosis of the left vertebral artery, and she had a surgical repair. In 2020, she had a workup for cholestatic disease including an abdominal MRI that showed a large pericardial effusion with pericardial thickening (Figure 2). The effusion was drained, and there were no malignant cells. Follow‐up cardiac MRI revealed right atrial wall thickening and an inflammatory locus. Systemic treatment of LHC was not given at this time as there was no biopsy proven data to suggest that it was causing her vascular issues. Cardiothoracic surgery was hesitant to perform a pericardial biopsy given the risk of the procedure, and her disease was stable.

**FIGURE 2:**
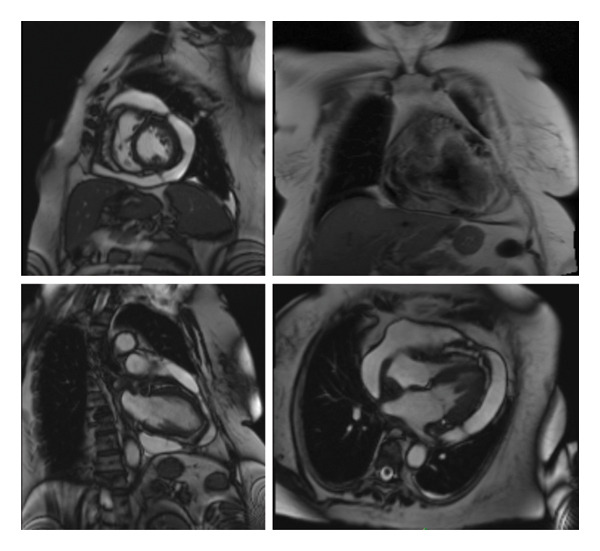
Cardiac MRI with evidence of soft tissue mass anterior and superior to right atrium and pericardial effusion. Obtained study in 2020. Cardiac MRI showing LVEF of 72% and a normal right ventricular systolic function. Moderate to large pericardial effusion seen with no evidence of tamponade or constrictive physiology. Imaging also shows thickening of right atrial walls anterosuperiorly with potential soft tissue mass anterior and superior to the right atrium suggestive of infiltrative processes, such as histiocytosis.

As LHC would be unlikely to have such extensive vascular involvement, the patient underwent a pericardial biopsy in 2024 which identified ECD (NexGen sequencing showed BRAF V600E positive, some other variants with potential clinical significance include ATM L752V 45.9% VAF, FGF 4R192Q at 47.5% VAF, MAP3K1 insertion at 45.1% VAF, MRE11A missense mutation of 44.9% VAF, SDHC missense mutation of 48.6% VAF). She was subsequently initiated on systemic therapy with vemurafenib (BRAF inhibitor). Unfortunately, she was unable to tolerate this after developing hair loss, unsteady gait, falls, ageusia, and neuropathy in her feet. Seven months later, after functional status was improved, she was started on dabrafenib and trametinib. She is tolerating well at this time and clinically improving.

## 3. Discussion

Histiocytosis encompasses LCH and non‐LHC. ECD is a rare form of non‐LHC. Mixed histiocytosis involving LCH and ECD is quite rare; however, there does appear to be an association in cases of simultaneously diagnosed ECD and LCH [8]. About 20% of patients with ECD have or will develop mixed histiocytosis [7]. It is important for clinicians to be aware of the association for timely and accurate diagnosis. Our case is an important example of new findings (cardiac involvement) not aligning with the original histiocytic disease, requiring further biopsy to evaluate for concomitant disease.

LCH and ECD can be treated w/interferon alpha treatment with response in 50% of patients [8]. One study found BRAF V600E mutation in 69% of LCH lesions and 92% of ECD lesions suggesting that there is a potential link between the two diseases and a BRAF V600E mutation, potentially as a progenitor cell origin. BRAF inhibitors could also be a potential treatment option [9]. Other less common mutations have also been identified, including MAP2k1, N/KRAS, ARAF, PIK3CA, and ERBB3 as potential mutations seen in ECD and LCH [8].

Regarding treatment options for ECD, corticosteroids and immunosuppressants were not associated with improved survival [9]. Given that ECD is multifocal requiring systemic treatment, surgical resection is not curative, and ECD is not sensitive to radiation except for palliative intent for tumors causing internal organ compromise [9]. For patients with BRAF V600‐mutant ECD with cardiac or neurologic involvement, such as our case patient, first‐line treatment would be BRAF inhibitors [9]. Next‐generation sequencing is recommended to evaluate for other MAPk‐ERK pathway alterations to determine if MEK inhibitors could be another targeted treatment option [9]. In patients with mixed histiocytosis, IFN‐alpha therapies could be suboptimal [9].

Once treatment is started, PET scans may be used to monitor response to therapy 3–6 months to assess metabolic response as well as organ‐specific CT and MRI imaging every 3–6 months [9]. After the disease stabilizes, imaging frequency can be decreased to every 6–12 months [9]. Given the nature of mixed histiocytosis, it is also recommended that patients undergo yearly evaluation for endocrinopathies, especially to evaluate pituitary hormones [9].

## 4. Conclusion

The case presented here is an example of the diagnostic challenge of a patient with two rare conditions. Although the prevalence of LHC and EC is low, one must keep the differential open to a combined disease process, especially when LHC is diagnosed first [4, 6]. It is important to recognize which organs are predominantly involved in the disease process and prompt further workup. Delay of diagnosis does impact care and decision‐making. In this case report, the diagnosis of ECD was delayed leading to delay in introduction of therapy. For treatment of mixed histiocytosis, the patient was initially tried on vemurafenib, which she was unable to tolerate due to significant side effects. The patient is currently undergoing systemic therapy with dabrafenib and trametinib with continued surveillance and has demonstrated clinical improvement.

## Funding

No funding was received for this manuscript.

## Consent

Written informed consent was obtained from the patient for this case report.

## Conflicts of Interest

The authors declare no conflicts of interest.

## Data Availability

The data that support the findings of this study are available from the corresponding author upon reasonable request.
